# Supplementary material for: Management of common minor ailments in Qatar: Community pharmacists’ self-perceived competency and its predictors
Source: PLoS One. 2021 Aug 16;16(8):e0256156. doi: 10.1371/journal.pone.0256156 (PMC8367001; doi:10.1371/journal.pone.0256156)
Supplement: Attachment — Submitted filename: Response to Reviewers-2.docx [file pone.0256156.s002.docx]

**Response to Review Comments**

27 July 2021

The Editor-In-Chief

PLOS ONE

Dear Sir,

Re: Manuscript ID PONE-D-21-08313R1 – “Management of common minor ailments in Qatar: Community pharmacists’ self-perceived competency and its predictors”

Our sincere thanks for the opportunity to revise the manuscript ID PONE-D-21-08313R1 – “Management of common minor ailments in Qatar: Community pharmacists’ self-perceived competency and its predictors” which is under your consideration for publication in PLOS ONE. We thank the reviewer and the editor for the insightful and useful comments and these have been used to revise the manuscript accordingly. Please find stated below our point-by-point response to all the comments.

**EDITOR’S COMMENTS**

Please review your reference list to ensure that it is complete and correct. If you have cited papers that have been retracted, please include the rationale for doing so in the manuscript text, or remove these references and replace them with relevant current references. Any changes to the reference list should be mentioned in the rebuttal letter that accompanies your revised manuscript. If you need to cite a retracted article, indicate the article’s retracted status in the References list and also include a citation and full reference for the retraction notice.

**Response:** The reference has been reviewed and found complete.

**REVIEWER’S COMMENTS**

**Reviewer #1:**

**Comment-1**) I am fine with the corrections made, except for the following:

I would advise against the approach you took in your rebuttal to reviewer comments. On several instances, the feeling appeared to be 'while we thank the reviewer for the comment, we did it the right way' and will disregard it.

**Response-1:** We are truly grateful for the valuable comments offered by the reviewer. We sincerely apologize for any perceived disregard of some of the comments. This was not our intention and we will never set out to do that. Perhaps, our desire to provide detailed explanations underlining some of our rebuttal may have accounted for this. We value the suggested corrections proposed by the reviewer and we are convinced it can only improve the scholarly value of the manuscript.

**Comment-2)** For example, regarding the Likert scale sequence, it will not suffice to say that responders were 'given detailed instructions' and things were successful. Likert scales are very tricky devices, and to ask for input into what came b/n Low and Full was a reasonable request to make.

**Response 2:** We totally concur with the reviewer that the request for explanation of the response scale used for the self-perceived competency assessment is reasonable. We did not disregard this request but just tried to explain how it was done as stated in the manuscript. In fact, the scale that we used in the study was not a Likert scale but a semantic differential scale which allowed respondents to choose from paired adjectives at the opposite end of a continuum (1 to 10) (1: low competence, 10: full competence). This has been made clear in the revised manuscript [line 175].

*“The participants ranked their responses to the 12 competency elements (condition- and non-condition specific) in Section B on a semantic differential scale of 1 to 10 (1: low competence, 10: full competence)”.*

**Comment-3)** Secondly, to state that Description and Signs/Symptoms were sufficiently distinct items, I would love to be enlightened. When I think of the Description of the Common Cold, I conjure up "a viral upper respiratory infection manifesting as nasal congestion, runny nose, perhaps a cough, maybe a headache etc. Now, how do signs/symptoms deviate from that?

**Response-3:** We thank the reviewer and concur that there are possibility of overlap between description and sign/symptoms. However, we are of the opinion that this does not obviate the difference between both items. We thought that a description/definition is generally an overview or broad characterization while signs/symptoms are usually more specific. For instance, a viral upper respiratory infection can be described as infection of the upper respiratory tract caused by common cold virus such as a rhinovirus, influenza virus or coronavirus. However, we concur that some people may proceed to add sign and symptoms to the definition / description.

**Comment-4)** For line 346, the statement -- "probably suggests the study findings are valid and reliable" -- based on a sole Cronbach's alpha is too far-reaching. I like your items in Table 3. Regardless of that stance, do you really feel you got things so right in the survey to make that conclusion? I look back to all the OTC research I have done over 30 years and I STILL worry about what mistakes I made.

**Response-4:** We concur with the reviewer that the risk of an over-reach is always an issue in survey research. The manuscript has been revised to ‘tone down’ the phrase to *“probably suggest that the study findings are valid”* [line 346].

**Comment-5)** Lastly, to downgrade "significantly add to the literature" to "may significantly add" is still clearly over-valuing the work you created. It is just not that profound, similar to the rest of us working in this area.

**Response-5:** We thank the reviewer for this suggestion. The manuscript has been revised as follow: *“may add to global knowledge in the study area”* [Line 339].
